# Optimization of range based self-localization problem in wireless sensor networks using improved cuckoo search algorithm

**DOI:** 10.1038/s41598-025-23405-0

**Published:** 2025-11-13

**Authors:** Srilakshmi Aouthu, Narra Dhanalakshmi, T. Seshagiri, Ravilla Dilli

**Affiliations:** 1 Electronics and Communication Engineering Department, Vasavi College of Engineering, Hyderabad, 500031 Telangana India; 2 Electronics and Communication Engineering, VNR Vignana Jyothi Institute of Engineering & Technology, Hyderabad, 500090 Telangana India; 3 Electronics and Communication Engineering, Sri Venkateswara College of Engineering & Technology, Chittoor, 517127 Andhra Pradesh India; 4 Electronics and Communication Engineering, Manipal Institute of Technology, Manipal Academy of Higher Education, Manipal, Udupi, 576104 Karnataka India

**Keywords:** Anchor node, Localization, Localization accuracy, Range-based localization, Wireless sensor network, Electrical and electronic engineering, Natural hazards

## Abstract

Self-localization is the capability of a wireless sensor network (WSN) to estimate the location coordinates of a given target node (TN), using the location knowledge of few anchor node (AN)s. The ANs are the sensor nodes installed with GPS modules, and their locations are known in prior. Precision improvement of the TN’s location is an important issue for effective data transmission in WSNs. Localization draws attention in location aware applications like nuclear attacks, object tracking, healthcare, supply chain management, biological attacks, and traffic monitoring, etc. In this article, a weight based AN selection strategy in localization algorithm for WSNs is proposed, which uses only four ANs where each one is installed with GPS module, and deployed within the sensing area. The weights are assigned to ANs depend on the distance between ANs and TNs to mitigate the effect of noise due to spatial and temporal changes in the sensing area. The proposed method uses improved cuckoo search algorithm, which is a nature inspired optimization technique to address the anisotropic nature of WSNs, and compute global optimal location coordinates of TNs. It also enhances the localization accuracy compared to the nascent localization algorithms in WSNs. Rigorous simulations are conducted to prove the efficiency of the proposed method using the performance metrics as number of ANs, mean localization accuracy, and communication range. Based on the simulation results, the localization accuracy of the proposed CERBLA algorithm is increased to 99.24% and the range measurement error is minimized to 1.18 m. The localization accuracy using the CERBLA algorithm is enhanced by 25.32%, 25.19%, 21.47%, 128.21%, 84.48%, and 181.35% compared to DCK-GWO, WOA-QT, DECPSODV-Hop, ECS-NL, QABA, and IDE-NSL-AWSN algorithms respectively. The performance of the CERBLA is outperforming the existing algorithms particularly, when the number of ANs are less than ten, and this feature is attractive to build cost-effective localization algorithms for indoor applications of WSNs.

**Supplementary Information:**

The online version contains supplementary material available at 10.1038/s41598-025-23405-0.

## Introduction

 The objective of localization in WSNs is to compute the exact location coordinates of each SN using a few ANs. The self-localization techniques in WSNs are broadly classified into “range-free” and “range-based” algorithms. Range-free localization schemes are hop-count based but not based on distance between SNs and ANs. These techniques are based on solutions to optimization or heuristic problems, and they are decentralized in nature. The advantage of these techniques is that they need fewer ANs to compute the location coordinates of unknown TN locations. As the SNs in WSNs are operated with limited hardware implementations, and energy resources, it is a good choice to select range-free localization algorithms for locating the SNs based on their connectivity in the network terrain. But these algorithms do not provide higher localization accuracy. The examples of these techniques are DV-hop, and Centroid. On the other hand, range-based localization techniques compute the Euclidean distances between the SNs and ANs, and locate the SNs using geometric techniques like TOA, TDOA, AOA, and RSSI^1–5^. These techniques provide higher localization accuracy. However, these techniques expect a greater number of ANs, which increases the cost of WSNs. The performance of any given localization technique is defined in terms of localization accuracy, computational complexity, energy consumption, and communication overhead^6–9^. Achieving localization accuracy is further challenging when the SNs are mobile in WSNs, and the static localization algorithms will fail to output the accurate location of SNs. The ANs which are equipped with GPS modules still have disadvantages particularly WSNs deployed in hard-to-reach areas as well as indoor applications. Therefore, it is essential to define the localization algorithms which are GPS information independent in computing SN locations.

## Contributions

### Motivation

In the proposed method, we have used only four ANs to localize the rest of the SNs in the network, using RSSI values of signals received from ANs. RSSI based localization is a cost-effective solution in WSNs, but most of the existing RSSI based localization algorithms perform efficiently in static scenarios. In the case of mobile SNs in the network, static localization algorithms refresh the localization process frequently that consumes higher energy and causes more localization errors. The existing range-based localization algorithms did not consider the anisotropy factors typically seen in WSNs, leading to poor positioning accuracy. Also, most of the existing localization schemes in WSNs select ANs randomly and they need a greater number of ANs for accurate location calculations of SNs, which increases the cost of WSNs. In this article, a low-complex range based self-localization algorithm that uses RSSI values is proposed to address the anisotropic nature of WSNs and achieve higher location accuracy. The existing CSO algorithm depends on the information exchange between populations to maintain the population diversity. In case of poor diversity, the CSO algorithm falls into local optima. We have proposed a true fitness function based on the distances between TNs and ANs to ensure the population diversity. Also, the conventional CSO updates the location and path through Lévy flight. Information exchange among populations is derived through communication between individuals and the best current population, which causes CSO to be susceptible to local optima and lower convergence accuracy. The proposed CERBLA improvised over the similar natural inspired algorithms used for localization where the signals from the best three anchor nodes are considered. The weighted centroid is used in locating TNs based on signals from ANs.

The main contributions presented in this article are as follows:

i. Rigorous literature study on the existing localization techniques and algorithms is conducted that enhance the localization accuracy in WSNs.
ii. For the first time, range-based localization algorithm is proposed that uses only four ANs. It exploits the co-planar properties of ANs to mitigate the localization errors.
iii. Computationally cost-effective fitness function is defined to achieve global optimal location coordinates of TNs. During the initial rounds of localization, the TNs which are localized are designated as assistant ANs. These SNs help in enhancing the coverage in WSNs.
iv. Intensive simulations are conducted to prove the efficiency of the proposed algorithm in terms average localization error and localization accuracy, by considering the metrics as node density and number of ANs.
v. The comparative analysis is performed under the similar network deployment conditions and localization task.

The remaining content of the article is structured as follows: Sect. 3 briefs out the nascent localization algorithms, methods, and techniques that exists in the literature, Sect. 4 presents methodology of the proposed technique, the simulation results and discussions are mentioned in Sect. 5, also the results of the proposed method are compared with the existing nascent localization algorithms, and finally Sect. 6 mentions the conclusions of the proposed algorithm.

## Related works

Increasing the number of ANs causes higher energy consumption and cost in WSNs, but introducing mobility in ANs leads to not only higher localization accuracy but also less energy consumption^10^. Accurate TOA estimation demands higher signal bandwidth of 500 MHz in RF-based WSN localization which is highly power inefficient and complex. However, TOS estimation based on IEEE 802.15.4 ZigBee^11^ needs a narrow bandwidth of 2 MHz, at the same time the localization accuracy of 0.5 m at SNR of 8dB. The TOA based localization algorithms expect precise time synchronization which is energy inefficient. Therefore, the TDOA localization is introduced in which the mutual distances between SNs are computed based on the difference in received signal arrival times. These signals are received from spatially separated receivers. TDOA based localization combines the “Multilateral Maximum Likelihood” and “Trilateration Method” to achieve optimized localization effect^12^. Using the SNs that interconnect TNs, a subset of SNs is created as a backbone. This subset is used as a reference to locate the other SNs which are not part of this subset^13^. Optimal AN pairs selection in range-free localization can enhance the location measurements accuracy in anisotropic WSNs. In anisotropic WSNs, reliable and optimal AN pair are defined to get high localization accuracy by deriving the distance between TN and AN. Estimation of this distance uses average hop progress for enhancing the ANs utilization^14^.

DV-Hop algorithm considers the hop distance between AN and the closest unknown TN as the global average hop distance. It does not consider the effect of hop count in defining the location coordinates of TN, and it leads to localization errors. The improved version of “minimum mean square” based DV-Hop localization algorithm^15^ considers the effect of different hop SNs in the vicinity of TN is considered in computing the SN coordinates and it significantly enhances the localization accuracy. DV-Hop algorithm considers the number of hops between TNs and ANs, the average distance per hop at AN is defined to estimate the location of TN. When the hop count is uniform, the communication range is fixed, and it leads to poor localization. To establish the relation between physical distance and hop count, different communication powers are maintained at different SNs. The TNs calculate the average distance per hop between itself and the three closest ANs and take the average of them to get the more accurate location values of the TNs^16]– [17^. The breakdown of AN during the localization process affects the accuracy in location measurements in multi-hop WSNs. Online sequential DV-hop localization algorithm will address this problem and minimize the average location errors^18^. Improved version of DV-hop localization algorithm based on TLBO and collinear properties of ANs can improve the convergence rate and precision of location measurements^19^. The triangular pyramid spaces constructed by neighbor ANs of the TN are divided into subspaces. The centroid of the subspace computes the location of TN with minimal error^20^. One more way of addressing the drawbacks of DV-hop algorithm is to use “half-measure weighted centroid”^21^, in which TNs realize their locations using centroid algorithm, and use this location accuracy as weights for locating TNs.

In RSSI based localization, the RSSI values decrease with increasing range and causes poor localization. However, in the presence of known environment and decision upon multiple scans, the impairments in accurate RSSI measurements are minimized^22^. AN selection is an important aspect in RSSI based localization to mitigate the errors in location measurements. “Weighted centroid localization”^23– 24^ algorithm is based on RSSI that enhances positioning accuracy by tuning the correction coefficient. Even though RSSI based localization is a cost-effective solution, the shadowing effects introduce high errors in range measurements. Methods like majority rule approach, centroid-based outlier detection, and mean-shift clustering for outlier detection minimizes localization error in shadowing environments^25^. In RSSI-based localization, ML techniques along with its CRLB save the SNs energy^26^, and communication bandwidth^27^. Nature-inspired optimization algorithms^28–31^ show the solutions to the critical issues like network coverage, energy efficiency, connectivity, and localization in WSNs.

The state-of-the-art localization meta-heuristic algorithms and methods^32–35^ like GA, PSO, ACO, BOA, FOA, FPO, GWO, ABCOA, FSO are proposed to optimize the localization errors. Defining virtual ANs for localizing the moving TNs using meta-heuristic algorithms like PSO and FOA minimize the need of physical ANs in WSNs^36–38^. The performance metrics to analyze the performance of these localization algorithms include convergence rate, accuracy, number of localized nodes, complexity, cost, energy consumption, and scalability. SNs redundancy in underwater WSNs causes low positioning accuracy. PSO based AN optimization improves the localization accuracy under the consideration of SN’s residual energy and communication overhead^39– 40^. Heuristic approach using algorithms like GA shows optimal trade-off between intensification and diversification. But, in NLOS environments, there exists discontinuities in the fitness function evaluation of SN distribution and challenges during the exploration. Hybrid GA which is “Memetic Algorithm” with a Local Search strategy improves exploring process. The combination of hybrid GA and memetic algorithm enhances the node localization by 14.2% compared to GA based optimization^41^.

To enhance the performance of DV-hop localization algorithm, binary PSO is used to generate subsets of ANs. Using these subsets, each AN computes mean-hop size during self-localization stage, and broadcasts it to the nearest TNs. The TNs localize themselves using these subsets instead of depending on all the ANs and this procedure enhances the localization accuracy^42^. MCB algorithm localizes the TNs by exploiting the SNs mobility, and compute the average value of current location samples, but the lower ANs density in WSNs cause more localization errors. To address this issue, “Grey model prediction” based MCB algorithm^43^ is proposed to enhance the localization accuracy. This algorithm uses past time intervals’ location values in predicting the current location samples. CSO algorithm defines the set of ANs with certain weights, and they serve the TNs to locate themselves^44– 45^. Cooperative localization in low density ANs environment causes poor localization accuracy in WSNs due to the redundant information. In those scenarios, source optimization is introduced where each SN computes its own CRLB and informs the same to neighbors. Same way, each SN collects the CRLB from neighbors along with azimuth angle, distance to compute the final location. This scheme is based on fuzzy comprehensive evaluation^46^, and it enhances localization accuracy by 19.4%. The combination of three meta-heuristic algorithms called DEEC, “Gradient Distance Elimination”, and “Gaussian Elimination” are used to define the hyper-heuristic optimization model^47^ for SN localization in WSNs. It gives lower localization error compared to DV-hop, weighted centroid, compensation coefficient, weighted hyperbolic algorithms. Fuzzy based localization uses a weighted centroid technique^48^, where the TN position is estimated with high accuracy, which helps in increasing throughput and energy efficiency. Centroid iterative ML estimation in localization divides the WSN into tiers based on number of hops with respect to sink node. This helps to select optimal cluster heads and detect SN anomalies^49^.

The nascent works^50^ on location optimization are considered that includes swarm intelligence, evolutionary, and metaheuristic algorithms. Localization evaluation criteria are analyzed in terms of time complexity, convergence speed, and localization accuracy. DV-Hop with triangulation^51^ and “Bat-SA algorithm” aimed at fast convergence, but it gives higher location errors. DV-Hop with RSSI information^52^ and “triangle centroid” enhances the location accuracy of TNs by 58%. In this method, the hop distance matching factor is defined to accurately obtain the hop size and weigh the fitness of each AN. “geometric constraint” and “optimized weights” features in DV-hop^53^ finds location coordinates of TNs with minimal errors. least squares method. But the execution time is increased from 0.36s to 0.61s. “Chaotic crested porcupine optimizer^54^” replaces the least squares method in finding the TNs location coordinates. But the authors have maintained 300 m as communication radius, which is very high in energy constrained sensor networks, also it uses 20% of ANs which leads to higher deployment cost. “Multi-objective salp swarm with fuzzy selection^55^” is integrated into DV-hop algorithm aiming at the 50% reduction in location errors. However, it expects 30 ANs and a communication radius of 40 m which again leads to an increase in the deployment cost and poor energy efficiency. “PSO-Amorphous^56^” provides minimal localization error at the cost of local optima, premature convergence, and lesser scalability. It also expects a higher number of beacon nodes. “Crow Search weighted centroid localization^57^” minimizes the location error compared to DV-hop at the cost of additional communication packets in beacon selection process. An improved PSO^58^ based location finding expects higher SNRs for optimized sensor deployment and works efficiently only under LOS environments. When the sensor node’s communication range is set to 18 m with an error upto10% and the network size of 400 nodes, it is shown that^59^ the anchor node positions ratio is increasing from 5 to 35%, the localization errors are reduced due to the opportunity that target nodes receive signals from many anchor nodes. Also, when the communication range is increased from 15 to 45 m, the localization error is slowly decreasing.

##  Proposed method

In the existing localization algorithms of WSNs, once the optimal set of localized SNs are defined, all the rest of TNs use this set to locate themselves. This leads to inefficient utilization of ANs and causes more localization errors. Also, these algorithms need higher number of iterations to reduce the location deviation errors of individual TNs, and it leads to more energy consumption at each SN. Therefore, selection of optimal and more favorable ANs enhances the localization accuracy as well as energy efficiency. Assigning weights for the ANs signals received at TNs will mitigate the location measurement errors, compared to considering all signals received from ANs present in the communication range. As the RSSI values-based distance measurements are simple and efficient to implement in WSNs, in this section, we have proposed a low-complex range-based localization algorithm that uses RSSI values in selecting ANs and achieve minimal localization error compared with the existing techniques. The following assumptions are made by defining the proposed methodology:

1. The radio channels are anisotropic.
2. SNs are homogeneous with respect to communication range and hardware.
3. The channel irregularities and noise are considered.
4. All computations regarding localization are performed at TN to enhance energy efficiency.

At the initial stage, the TNs within the communication range of ANs calculate their own locations based on the RSSI values received from ANs. To perform localization, each TN needs a minimum of three RSSI values of signals received from various ANs. If the TN receives more than three signal RSSI values from ANs, then the TN chooses the best three RSSI values to calculate its location coordinates. We have proposed a localization technique by defining the fitness function as mentioned in Eq. 1 that involves range or distance measurements. The accurate location coordinates are achieved at TN by minimizing the fitness function values as shown in Eq. 1.

1 $$\:\mathrm{F}\left({\mathrm{x}}_{\mathrm{m}},\:{\mathrm{y}}_{\mathrm{m}}\right)=\:\frac{1}{\mathrm{N}}\sum\:_{\mathrm{m}=1}^{\mathrm{N}}{({\mathrm{d}}_{\mathrm{m}\mathrm{n}}-{\mathrm{d}}_{\mathrm{m}\mathrm{n}}^{1})}^{2}$$

Where $\:\mathrm{F}\left({\mathrm{x}}_{\mathrm{m}},\:{\mathrm{y}}_{\mathrm{m}}\right)\:$is the MSE between the TN whose location coordinates are computing and all ANs present in the communication range of the given TN. $\:\left({\mathrm{x}}_{\mathrm{m}},\:{\mathrm{y}}_{\mathrm{m}}\right)$ are the location coordinates of TN and $\:\left({\mathrm{x}}_{\mathrm{n}},\:{\mathrm{y}}_{\mathrm{n}}\right)$are the location coordinates of AN. $\:{\mathrm{d}}_{\mathrm{m}\mathrm{n}}$ and $\:{\mathrm{d}}_{\mathrm{m}\mathrm{n}}^{1}$ are the actual distance and the estimated distance between TN and AN respectively. The estimated distance between TN and AN is modelled using Eq. 2.

2 $$\:{\mathrm{d}}_{\mathrm{m}\mathrm{n}}^{1}=\:{\mathrm{d}}_{\mathrm{m}\mathrm{n}}+{\mathrm{e}}_{\mathrm{m}\mathrm{n}}$$

where $\:{\mathrm{e}}_{\mathrm{m}\mathrm{n}}\:$is the error in computing range between TN and SN. In the presence of LOS path between TN and AN, the free-space path loss model is used to calculate RSSI values as shown in Eq. 3.

3 $$\:{\mathrm{R}\mathrm{S}\mathrm{S}\mathrm{I}}_{\mathrm{d},\:\mathrm{L}\mathrm{O}\mathrm{S}}={\mathrm{G}}_{\mathrm{T}}{\mathrm{G}}_{\mathrm{R}}{\mathrm{P}}_{\mathrm{T}}({\frac{{\uplambda\:}}{4{\uppi\:}\mathrm{d}})}^{2}$$

where $\:{\mathrm{G}}_{\mathrm{T}}$ and $\:{\mathrm{G}}_{\mathrm{R}}\:$are the antenna gains at AN and TN respectively. $\:{\mathrm{P}}_{T}$ is the transmitting signal power at AN. ‘d’ is the distance between TN and AN, and ‘$\:{\uplambda\:}$’is the signal wavelength. In case of NLOS path between TN and AN, RSSI values are computed using log-normal path loss model as shown in Eq. 4 that accounts the shadowing and multipath effects of signals propagation in the radio channel.

4 $$\:{\mathrm{R}\mathrm{S}\mathrm{S}\mathrm{I}}_{\mathrm{d},\:\mathrm{N}\mathrm{L}\mathrm{O}\mathrm{S}}={\mathrm{P}}_{\mathrm{T}}-10\mathrm{a}{\mathrm{l}\mathrm{o}\mathrm{g}}_{10}\left(\frac{\mathrm{d}}{{\mathrm{d}}_{0}}\right)-\mathrm{P}\mathrm{L}\left({\mathrm{d}}_{0}\right)+{\mathrm{X}}_{{\upsigma\:}}$$

$\:\mathrm{P}\mathrm{L}\left({\mathrm{d}}_{0}\right)$ is the path loss at reference distance $\:{\mathrm{d}}_{0}$. ‘a’ is the path loss exponent which varies between 2 and 4 according to environmental conditions. $\:{\mathrm{X}}_{{\upsigma\:}}$ is the gaussian noise whose “mean” is zero and standard deviation is ‘$\:{\upsigma\:}$’. The variance ‘$\:{{\upsigma\:}}^{2}$’ is proportional to the square of true distance $\:{\mathrm{d}}_{\mathrm{m}\mathrm{n}}$ between $\:{\mathrm{m}}^{\mathrm{t}\mathrm{h}}$ TN and $\:{\mathrm{n}}^{\mathrm{t}\mathrm{h}}$AN as shown in Eq. 5.

5 $$\:{{\upsigma\:}}^{2}={\mathrm{d}}_{\mathrm{m}\mathrm{n}}^{2}\times\:{{\upeta\:}}^{2}$$

where ‘$\:{\upeta\:}$’ is the distance error factor. In the real applications of WSNs deployment, larger distances between SNs leads to more localization errors. To reduce these location measurement errors and improve the localization accuracy, an optimal AN selection strategy is proposed based on RSSI values. The proposed algorithm presented in this section has mainly three stages: identification & classification of TNs, optimal selection of ANs, and finally, accurate range estimation to enhance the localization efficiency.

### TNs identification & classification

The RSSI values of signals received from ANs are measured at each TN for LOS and NLOS paths using Eqs. 3 and 4 respectively. Calculate the difference between maximum and minimum of the measured RSSI values as RSSI_diff_. This value is compared with a threshold distance to decide whether the given TN is either a boundary or non-boundary node as per the Eq. 6. If RSSI_diff_ value is above the threshold distance, then the corresponding TN is decided as a boundary SN, otherwise it is a non-boundary SN.

6 $$\forall ,TN_{K} = \left\{ \begin{gathered} boundary\,SN,\,\,\,\,\,\,\,\,\,\,\,\,\,\,\,\,\,RSSI_{{diff}} \ge D_{{th}} \hfill \\ non - boundary\,SN,\,\,\,\,otherwise \hfill \\ \end{gathered} \right.$$

where

7 $$D_{{th}} = 0.35 \times \left| {\max \left( {RSSI\left( k \right)} \right)} \right|$$

Usually, if the distance between TN and AN is maximum, then the corresponding RSSI value is smaller and vice versa. The value 0.35 in Eq. 7 is chosen as a scaling factor to define the optimal threshold value.

**Fig. 1:**
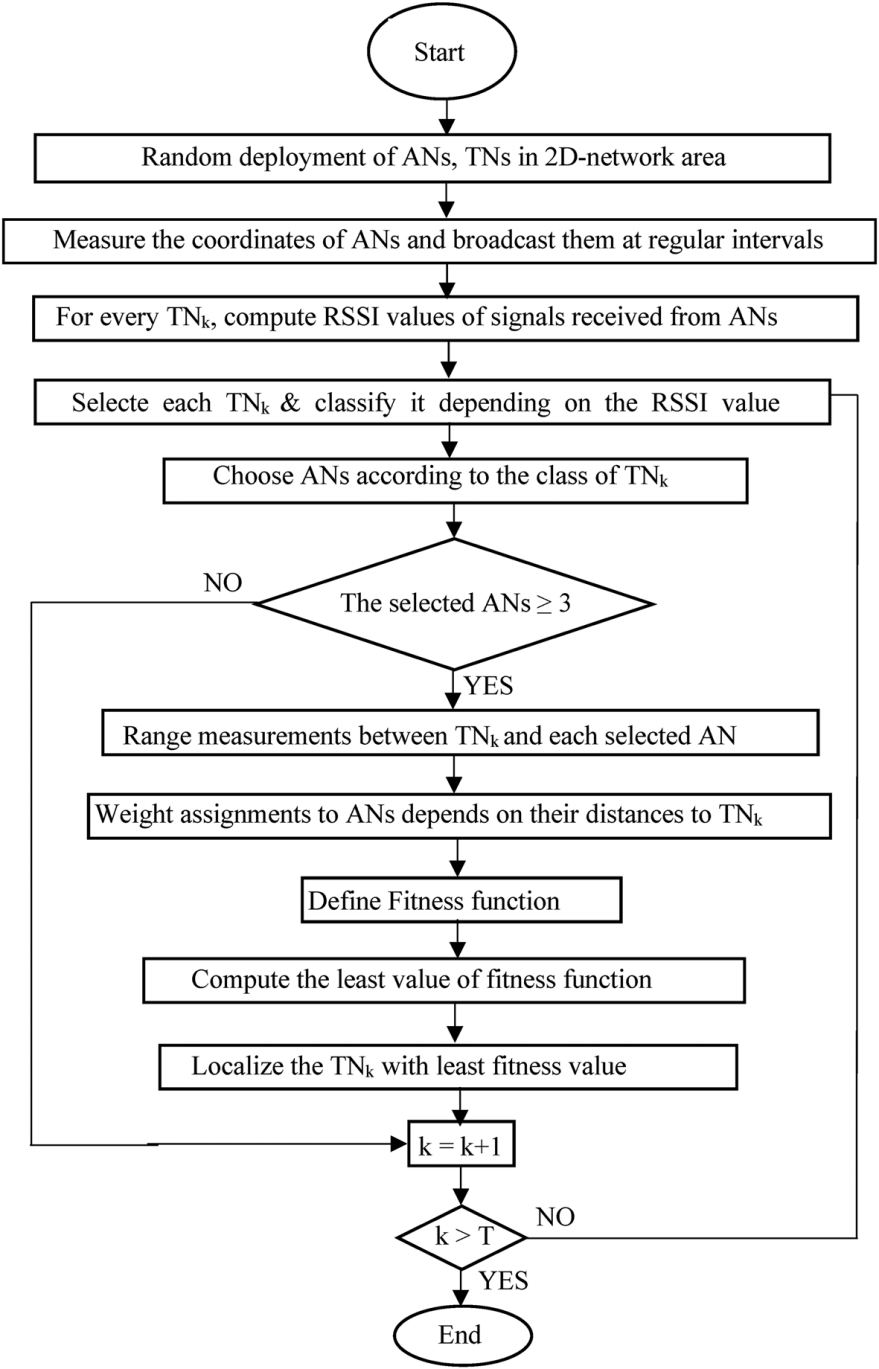
Flow diagram of the proposed CERBLA algorithm.

### Optimal selection of ANs

The RSSI values of the signals received from ANs are highly dependent on temporal and spatial changes in the wireless channel. Accounting the signals received from farther ANs in computing locations of TNs lead to more localization error. The focus at this stage is to define more favorable ways to improve the localization accuracy by reducing noise in the received signals from the selected ANs. To achieve this, an optimal ANs selection strategy is proposed which depends on whether the given TN is a boundary SN or a non-boundary SN. In the case of a boundary TN, identify three highest RSSI values and select the corresponding ANs in locating the given TN. In the case of a non-boundary TN, consider all the RSSI values and select all the corresponding ANs present within TN’s communication area. After conducting rigorous simulations, we define 0.8 as the proportional factor for a boundary TN to minimize the effect of errors due to the farther ANs on localization accuracy.

### Accurate location estimation

As shown in the flow diagram (depicted in Fig. 1) of the proposed method, we have considered atleast three ANs in computing the signal RSSI values and the location values. For achieving highest localization accuracy, the weights are assigned for the measured distances between every TN and the chosen ANs set. The assigned weights are inversely proportional to the measured distances and these weights are normalized as per Eq. 8.

8 $$\:{W}_{n}=\frac{{\left[{d}_{mn}^{1}\right]}^{-1}}{\sum\:_{n=1}^{{N}_{AN}}{\left[{d}_{mn}^{1}\right]}^{-1}}$$

$\:{W}_{n}$ is the weight assigned for n^th^ AN, $\:{d}_{mn}^{1}$ is the distance between m^th^ TN and n^th^ AN. $\:{N}_{AN}$ is the minimum number of ANs selected for location calculations. The contribution of AN in computing locations is less when it is much away from TN. The optimal fitness function is proposed to calculate the location coordinates as shown in Eq. 9. To achieve lower MSE of localization, the fitness function values should be less.

9 $$\:F\left({x}_{m},{y}_{m}\right)=\frac{1}{{N}_{AN}}\:\sum\:_{m=1}^{{N}_{AN}}{W}_{K}\times\:{({d}_{mn}-{d}_{mn}^{1})}^{2}$$

We have considered the optimization of localization in WSNs using improved CSO algorithm which is defined based on brood parasitism of cuckoo species. In improved CSO algorithm, new solutions are generated by either probability fraction ‘$\:{P}_{m}$’ of the total host nests or the Lévy flight (it is a random walk model with Lévy or power-law distribution with a heavy tail for the step-length). The best solution of global search in CSO algorithm depends mainly on mutation probability and the step size control factor. Keeping constant values for these parameters minimizes the convergence rate and localization accuracy in WSNs. Therefore, we have defined dynamic values for mutation probability and Lévy flight using Eqs. (10) and (11) in order to achieve global optimal solution with high localization accuracy.

Firstly, we define the mutation probability $\:{P}_{m}$ based on the fitness of solutions to overcome the problem of local convergence and enhance the population diversity. If we choose $\:{P}_{m}$ as too small value, localization accuracy is reduced and it needs more number of iterations to achieve optimal performance. On the other hand, if $\:{P}_{m}$ is too large, convergence time is more. Therefore, it is essential to have an optimal values of mutation probability that is proportional to the objective function value and it is defined using Eq. (10).

10 $$\:{\mathrm{P}}_{\mathrm{m}}\left(\mathrm{i}\right)=\:\left\{\begin{array}{c}\left({\mathrm{P}}_{{\mathrm{m}}_{\mathrm{m}\mathrm{i}\mathrm{n}}}+\:{(\mathrm{P}}_{{\mathrm{m}}_{\mathrm{m}\mathrm{a}\mathrm{x}}}-{\mathrm{P}}_{{\mathrm{m}}_{\mathrm{m}\mathrm{i}\mathrm{n}}}\right)\times\:\left(\mathrm{f}\mathrm{i}\mathrm{t}\mathrm{n}\mathrm{e}\mathrm{s}\mathrm{s}\left(\mathrm{i}\right)-{\mathrm{f}}_{\mathrm{m}\mathrm{i}\mathrm{n}}\right)\:\:if\:\left(\mathrm{f}\mathrm{i}\mathrm{t}\mathrm{n}\mathrm{e}\mathrm{s}\mathrm{s}\left(\mathrm{i}\right)-{\mathrm{f}}_{\mathrm{m}\mathrm{i}\mathrm{n}}\right)<1\:\\\:\frac{\:{\mathrm{P}}_{{\mathrm{m}}_{\mathrm{m}\mathrm{a}\mathrm{x}}}}{{\mathrm{N}}_{\mathrm{i}}},\:\:\:\:\:\:\:\:\:\:\:\:\:\:\:\:\:\:\:\:\:\:\:\:\:\:\:otherwise,\:\:where\:i=1,\:2,\:3,\:\dots\:\dots\:\dots\:.s\end{array}\right.\:\:\:\:$$

$\:{f}_{min}$ is the current global optimal fitness value and $\:fitness\left(i\right)$ is the current fitness value of i^th^ solution. $\:\left(fitness\left(i\right)-{f}_{min}\right)$ represents the quality of i^th^ solution. $\:{P}_{{m}_{min}}$ and $\:{P}_{{m}_{max}}\:$are the minimum and maximum values of the mutation probabilities respectively.

**Algorithm 1:**
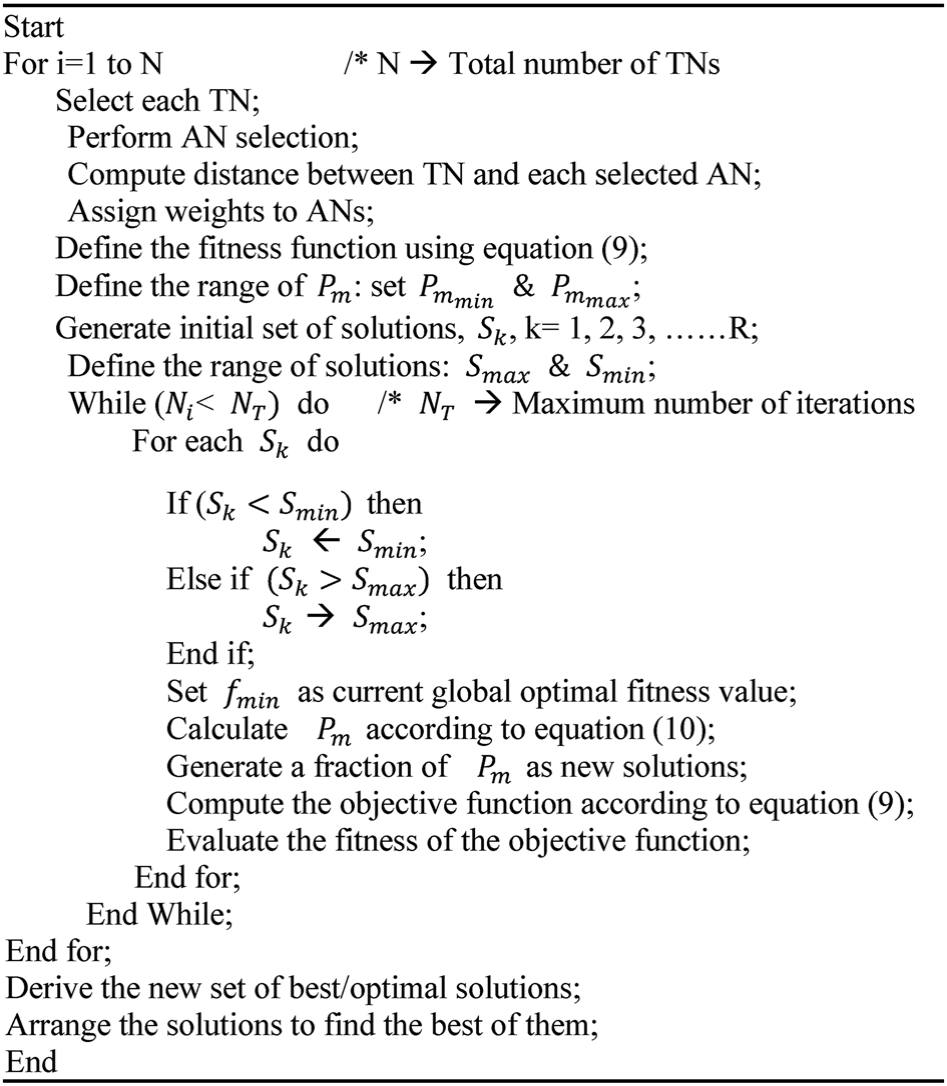
Algorithm of the proposed CERBLA protocol.

Secondly, the Lévy flight based solution which is a most efficient way of exploring the search space and new solutions are generated as

11 $$s_{i}^{{\left( {k + 1} \right)}} = s_{i}^{k} + \Delta .Le^{\prime}vy\left( \gamma \right)$$

where $Le^{\prime}vy\left( \gamma \right)$ is the step size and it is defined as $Le^{\prime}vy\left( \gamma \right) = \frac{x}{{\left| y \right|^{{\gamma ^{{ - 1}} }} }}$. $\:\gamma\:$ is a constant that takes values as $\:1<\gamma\:\le\:3$ and $\:\varDelta\:$ is the step size control factor. x and y are the normal distributions as $x\sim N\left( {0,\sigma _{x}^{2} } \right),~y\sim N\left( {0,\sigma _{y}^{2} } \right)$ with corresponding variances are given using Eq. (12).

12 $$\:{\sigma\:}_{x}={\left[\frac{{\Gamma\:}\left(1+\gamma\:\right)\mathrm{s}\mathrm{i}\mathrm{n}\left(\frac{\pi\:\gamma\:}{2}\right)}{{\Gamma\:}\left[\frac{1+\gamma\:}{2}\right]\gamma\:{2}^{(\gamma\:-1)/2}}\right]}^{{\gamma\:}^{-1}},\:{\sigma\:}_{y}=1$$

When the step size control factor $\:\varDelta\:$ is less, few of the newly generated solutions shown in Eq. (11) walk around the existing best solution to speed up the local search. On the other hand, when $\:\varDelta\:$ is high, the new solutions walk away from the present best solution and causes the absence of global optimal solution. Therefore, it is essential to define the variable step size as shown in Eq. (13).

13 $$\:\varDelta\:={\varDelta\:}_{max}-\frac{{N}_{i}}{{N}_{T}}\:({\varDelta\:}_{max}-{\varDelta\:}_{min})$$

where $\:{N}_{T}$ and $\:{N}_{i}$ are the total number of iterations and present iteration number respectively. $\:{\varDelta\:}_{min}$ and $\:{\varDelta\:}_{max}$ are the minimum and maximum step sizes respectively. From the Eq. (13), the size of $\:\varDelta\:$ decreases when the iteration numbers are increasing. During the initial iterations, the higher step sizes maximize the global search and at a later stage the lower step sizes intensifies the local search. Overall, we can achieve global optimal solution and higher conversion rate with enough number of iterations.

As WSN has energy constrained devices, it is essential to analyse the computational complexity of the localization algorithms. The computational complexity analysis is conducted to find the worst-case execution and completion times. If ‘s’, ‘a’, ‘g’, ‘i’, ‘d’, and ‘p’ are the total number of sensor nodes, anchor node, generations, iterations, spatial dimension, and population respectively, then the computational complexity of various techniques are analysed and compared as shown in Table 1. Though DV-Hop algorithm is simple and robust, it may not be suitable for locating SNs due to its higher localization error. Attempts were made to integrate DV-Hop and CSO for achieving precise location values^60^, but the execution time is higher. Also, the time complexity of DV-Hop algorithm is O((n-m)^4^) where ‘n’ total number of sensor nodes and ‘m’ is the number of anchor nodes. To increase the localization accuracy, an improved CSO is introduced^61^ having features of population grouping and drifting strategy (used for information exchange and population update), but the localization errors are up to 22.21%. The ANs ratio is 30%^61^ and 40%^60^ which leads to higher deployment cost of WSN. Its time complexity is O (T ∗ Nd ∗ D) where ‘T’, “Nd” and ‘D’ are the number of iterations, number of populations, and the spatial dimension respectively. Also, the communication radius maintained is about 30 m^60^ and 40 m^61^ which needs higher transmitting powers causing the faster depletion of sensor node’s battery.

**Table 1:** Computational complexity comparison.

Algorithm	Computational complexity
NDV-Hop^56^	O(a^2^)
NDV-Hop CSO^56^	O(g.p.(s–a))
DV-Hop	O(a^2^)
DV-Hop CSO^58^	O(g.p.(s–a))
DV-Hop PSO^59^	O(g.p.(s–a))
ICS-GD^57^	O (i. p.d)
Dragonfly algorithm^60^	O(s^2^)
Optimized Base Station location^60^	O(s. log(s))
CERBLA [proposed]	O(s.a)

## Results and discussions

**Fig. 2:**
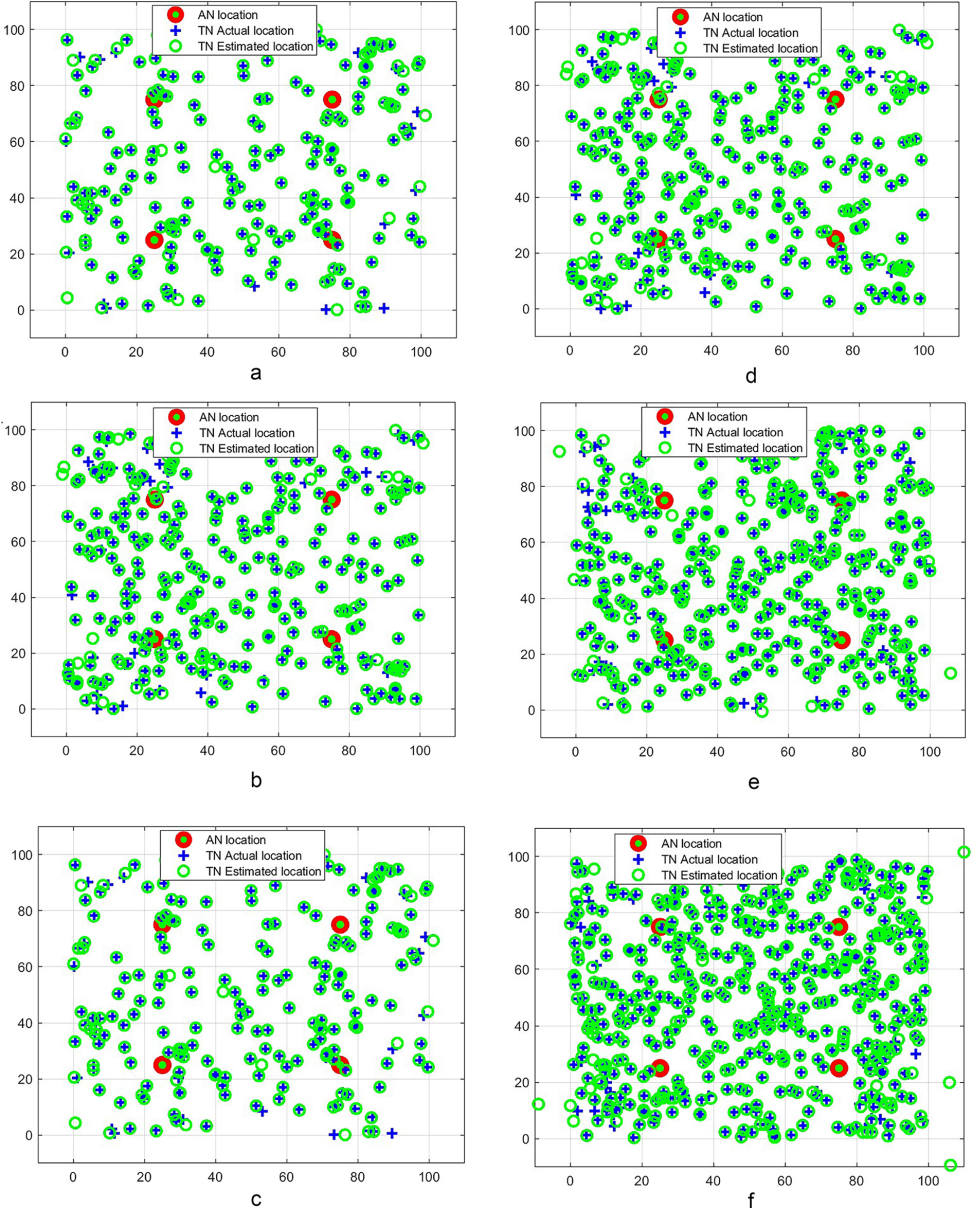
(**a**) Ideal case of localization for 100 SNs in 100 m$\:\times\:$100 m area. (**b**). Practical case of localization for 100 SNs in 100 m$\:\times\:$100 m area.**c** Practical case of localization for 200 SNs in 100 m$\:\times\:$100 m area.(**d**) Practical case of localization for 300 SNs in 100 m$\:\times\:$100 m area. (**e**) Practical case of localization for 400 SNs in 100 m$\:\times\:$100 m area. (**f**) Practical case of localization for 500 SNs in 100 m$\:\times\:$100 m area.

**Fig. 3:**
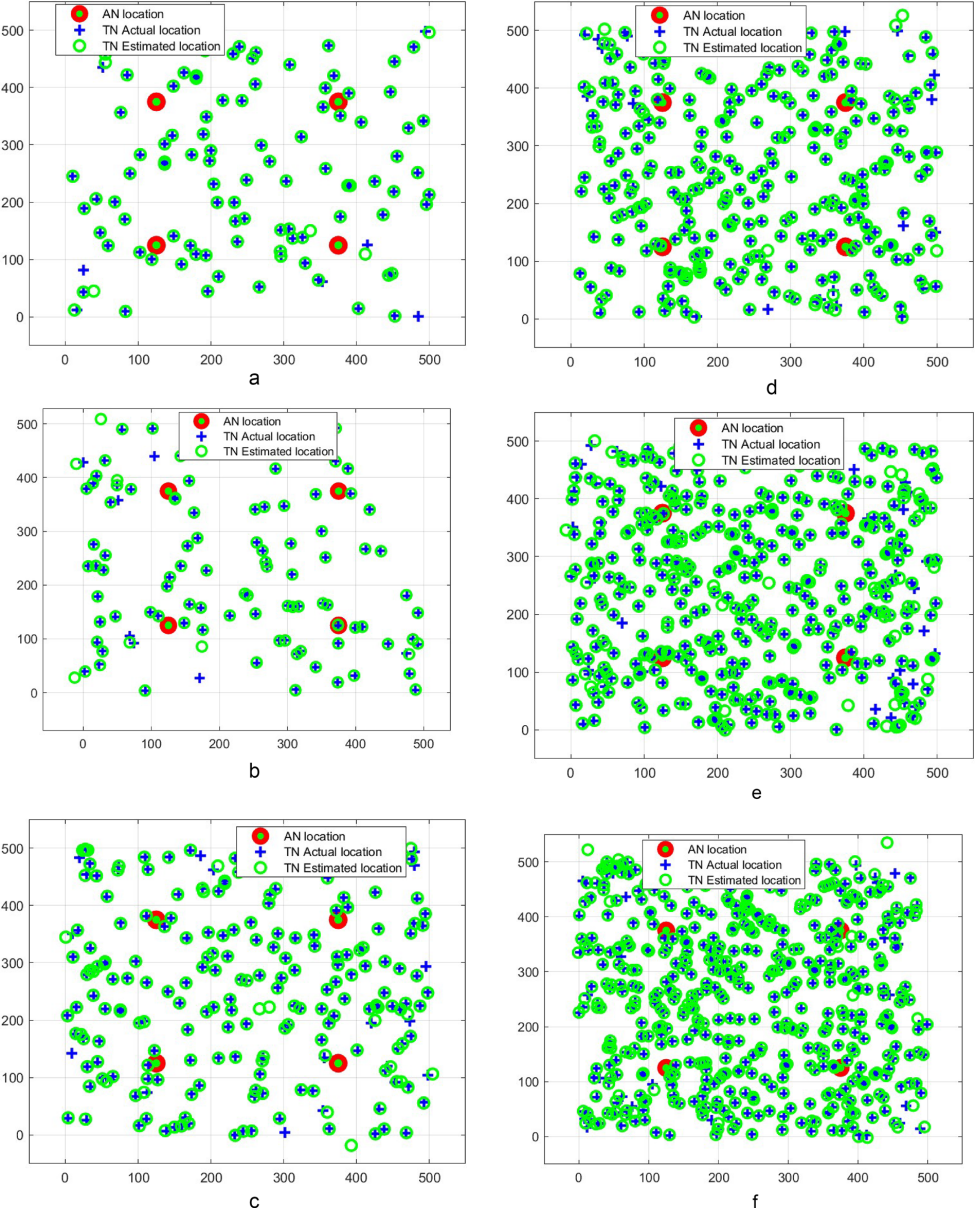
(**a**) Ideal case of localization for 100 SNs in 500 m$\:\times\:$500 m area. (**b**) Practical case of localization of 100 SNs in 500 m$\:\times\:$500 m area. (**c**) Practical case of localization for 200 SNs in 500 m$\:\times\:$500 m area. (**d**) Practical case of localization for 300 SNs in 500 m$\:\times\:$500 m area. (**e**) Practical case of localization for 400 SNs in 500 m$\:\times\:$500 m area. (**f**) Practical case of localization for 500 SNs in 500 m$\:\times\:$500 m area.

The performance of the proposed CERBLA is analyzed by conducting rigorous simulations in MATLAB 2024b environment. The SNs are deployed randomly as shown in Figs. 2a and f and 3a to 3f and the other simulation parameters are shown in Table 2. We have used only four static ANs and they are deployed inside the network terrain with 25 m and 125 m distance away from the corners of the network dimensions for $\:100\:m\:\times\:\:100\:m$ and $\:500\:m\:\times\:\:500\:m\:$ network sizes respectively. Mean localization error and mean localization accuracy are the performance metrics used to assess the performance of CERBLA. The performance comparison is conducted between the proposed CERBLA and the nascent localization algorithms. In Figs. 2a and 3a, the true locations and the measured locations of TNs are depicted using the CERBLA under the ideal scenarios of localization. In Figs. 2b and f and 3b to f, practical location measurements are presented under noisy conditions. In all cases, the location measurements using CERBLA are very close with their true locations, particularly when the node density is higher.

**Table 2:** Simulation parameters of the proposed CERBLA protocol.

Parameter	Value
Network area dimensions	100 m $\:\times\:$ 100 m, 500 m $\:\times\:\:$500 m
Number of SNs	100–500
Initial energy (E_0_)	0.5 J
Number of anchor nodes	4
Node density	0.001 m^2^ to 0.03 m^2^
Maximum communication range, R	50 m
Free-space energy (E_fs_)	50 nj/bit
Energy to operate the electronic circuity (E_elec_)	0.0013 pj/bit/m^4^
Energy consumption by amplifier (E_amp_)	10 pj/bit/m^2^
Size of data packet	4000-bits
Number of iterations	10,000
Number of simulations per measurement	20

The mean localization error is calculated as.

14 $$Mean{\text{ }}localization{\text{ }}error = \frac{{\mathop \sum \nolimits_{{i = 1}}^{N} \sqrt {\left( {x_{m} - x_{n} } \right)^{2} + \left( {y_{m} - y_{n} } \right)^{2} } }}{N}$$

In Eq. 14, ‘N’ represents the number of TNs. $\:({\mathrm{x}}_{\mathrm{m}},{\mathrm{y}}_{\mathrm{m}})$ and $\:\left({\mathrm{x}}_{\mathrm{n}},{\mathrm{y}}_{\mathrm{n}}\right)\:$are the true location coordinates and estimated location coordinates of the TN respectively.

**Fig. 4:**
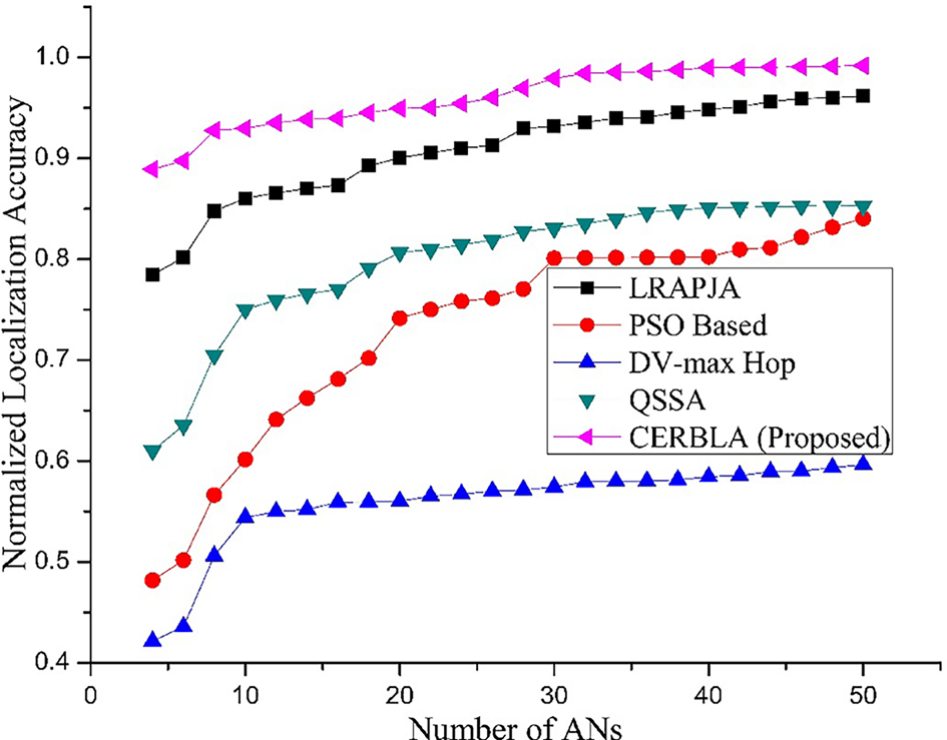
Comparison of Normalized localization accuracy versus number of Anchor nodes.

DV-Hop algorithm provides accurate location estimation in isotropic WSNs. DV-max Hop^65^ is a variant of DV-Hop algorithm meant for anisotropic WSNs to achieve higher location accuracy at minimal computational and communication overhead. It offers faster convergence at lower energy consumption. Range-free localization techniques are suitable for large-scale WSNs due to their low-cost hardware implementation. The range-free algorithms utilize only the neighborhood connectivity information in measuring the location of SNs. On the other hand, these techniques give poor localization accuracy and coverage in anisotropic WSNs. The combination of hop-based and geometric features can increase the localization accuracy in range-free localization by achieving trade-off between AN utilization and location measurements. PSO based^66^ location measurements of TNs enhance the mean localization accuracy by 31.4% compared to DV-max Hop algorithm. Range-free localization algorithm called LRAPJA is proposed that combines the geometric and hop based features of SNs to address the anisotropic nature of WSNs^67^. The anisotropic factors in WSNs limit the location precision of range-free localization algorithms. RAPS and QSSA techniques can enhance the localization accuracy in anisotropic WSNs^68^. Even in the presence of lower number of ANs particularly for less than ten, the localization accuracy is higher using the proposed CERBLA technique compared to other range-free and hop based algorithms as shown in Fig. 4. The proposed algorithm assures minimum of 88.92% localization accuracy in the presence of at least four ANs in the network and the accuracy improves further with the number of ANs up to 99.18% when 50% of SNs are act as ANs as shown in Table 3.

**Table 3:** Normalized localization accuracy versus number of anchor Nodes.

Number of ANs	Normalized Localization Accuracy				
	LRAPJA	PSO Based	DV-max Hop	QSSA	CERBLA (Proposed)
4	0.7848	0.4818	0.4216	0.6104	0.8892
6	0.8016	0.5018	0.4359	0.6352	0.8975
8	0.8478	0.5664	0.506	0.7048	0.9276
10	0.8604	0.6016	0.5441	0.7502	0.9297
12	0.8658	0.6412	0.5498	0.7595	0.9352
14	0.8701	0.6625	0.5519	0.7658	0.9386
16	0.8732	0.6812	0.5592	0.7702	0.9397
18	0.8924	0.7019	0.5595	0.7912	0.9452
20	0.9005	0.7416	0.5601	0.8068	0.9495
22	0.9055	0.7501	0.5652	0.8096	0.9501
24	0.9102	0.7584	0.5672	0.8142	0.9542
26	0.9127	0.7615	0.5702	0.8192	0.9601
28	0.9296	0.7705	0.5714	0.8275	0.9697
30	0.9318	0.801	0.5742	0.8306	0.9792
32	0.9357	0.8015	0.5794	0.8352	0.9842
34	0.9397	0.8017	0.5798	0.8402	0.9854
36	0.9407	0.8018	0.5801	0.8464	0.9862
38	0.9455	0.802	0.5814	0.8492	0.9876
40	0.9485	0.8024	0.5845	0.8505	0.9898
42	0.9512	0.8096	0.5856	0.8515	0.9902
44	0.9562	0.8115	0.5895	0.8518	0.9904
46	0.9594	0.8218	0.5901	0.8526	0.9906
48	0.9602	0.8317	0.5935	0.8525	0.9912
50	0.9616	0.8404	0.5964	0.8528	0.9918

**Fig. 5:**
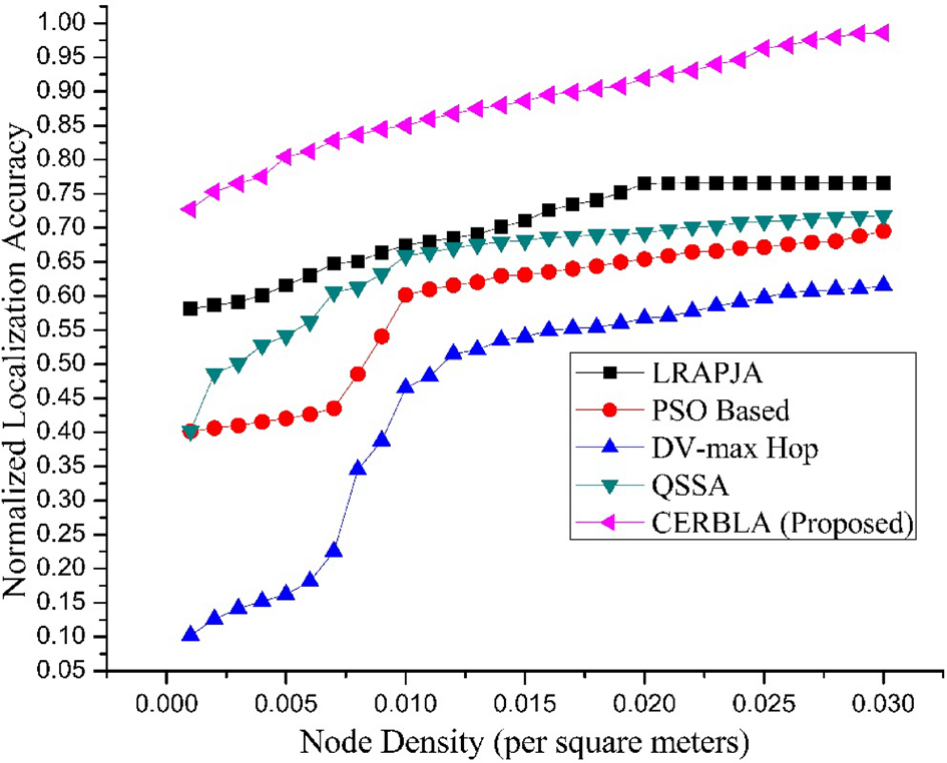
Comparison of Normalized localization accuracy versus Node density.

From the results shown in Fig. 5, hop based localization gives poor accuracy in the low node density conditions compared to meta-heuristic based localization algorithms. The proposed CERBLA algorithm outperforms the nascent localization algorithms due to its low complex nature of the fitness function. From Table 4, the higher localization accuracy is achieved using the proposed CERBLA algorithm even at the lower node densities compared to the hop-based localization and other meta-heuristic algorithms. However, the proposed CERBLA gives maximum localization accuracy of 98.62% at the node density of 0.03 per m^2^.

**Table 4:** Normalized localization accuracy versus node density (per m^2^).

Node Density (per m^2^)	Normalized Localization Accuracy				
	LRAPJA	PSO Based	DV-max Hop	QSSA	CERBLA (Proposed)
0.001	0.5814	0.4014	0.1024	0.4024	0.7274
0.002	0.5864	0.4064	0.1261	0.4864	0.7528
0.003	0.5914	0.4102	0.1416	0.5012	0.7649
0.004	0.6012	0.4156	0.1521	0.5276	0.7751
0.005	0.6155	0.4204	0.1624	0.5415	0.8042
0.006	0.6305	0.4264	0.1815	0.5624	0.812
0.007	0.6476	0.4354	0.2254	0.6056	0.8278
0.008	0.6506	0.4856	0.3452	0.6126	0.8364
0.009	0.6642	0.5409	0.3878	0.6325	0.8447
0.010	0.6745	0.6015	0.4652	0.6592	0.8498
0.011	0.6802	0.6096	0.4824	0.6642	0.8596
0.012	0.6852	0.6159	0.5152	0.6706	0.8675
0.013	0.6905	0.6203	0.5212	0.6754	0.8748
0.014	0.7014	0.6297	0.5355	0.6792	0.8799
0.015	0.7105	0.6309	0.5402	0.6816	0.8859
0.016	0.7259	0.6354	0.5492	0.6862	0.8952
0.017	0.7341	0.6401	0.5524	0.6874	0.899
0.018	0.7405	0.6439	0.5542	0.6896	0.9042
0.019	0.7518	0.6497	0.5601	0.6902	0.9078
0.020	0.765	0.6542	0.5674	0.6925	0.9175
0.021	0.7652	0.6589	0.5705	0.6976	0.9257
0.022	0.7654	0.6648	0.5772	0.7017	0.9305
0.023	0.7655	0.6659	0.5852	0.7025	0.9398
0.024	0.7656	0.6705	0.5916	0.7079	0.9461
0.025	0.7657	0.6714	0.5978	0.7098	0.9635
0.026	0.7658	0.6759	0.6052	0.7106	0.9678
0.027	0.7659	0.6786	0.6067	0.7134	0.0752
0.028	0.7659	0.6804	0.6095	0.7154	0.9798
0.029	0.766	0.6879	0.6105	0.7167	0.9851
0.030	0.7661	0.6952	0.6152	0.7182	0.9862

**Fig. 6:**
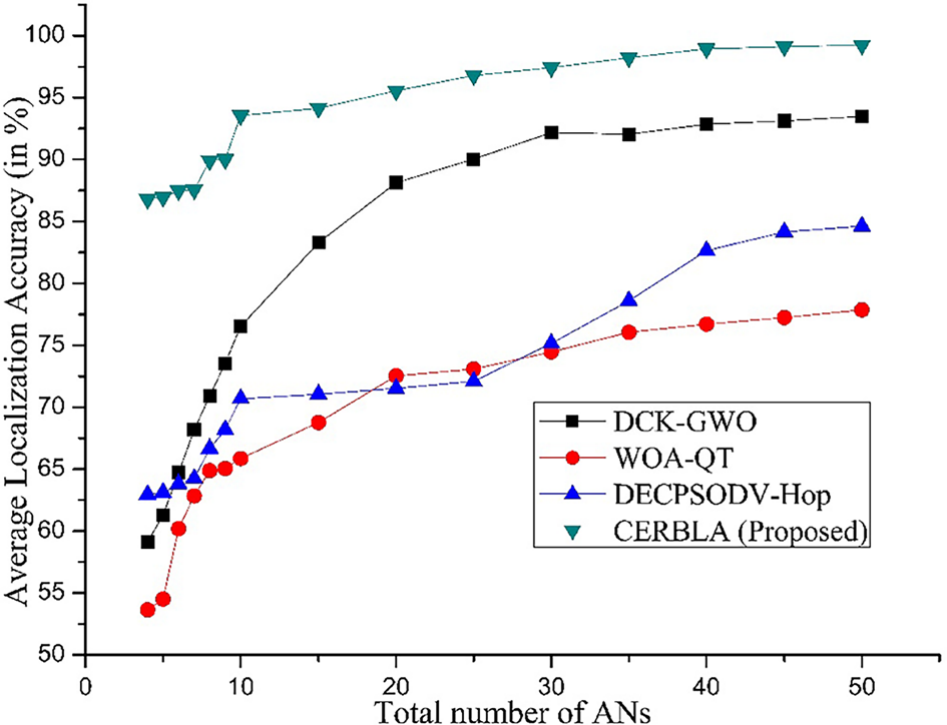
Average localization accuracy vs. number ANs.

The performance of average localization accuracy vs. ANs is compared between the proposed CERBLA and heuristic approach-based localization techniques in Fig. 6. DCK-GWO^69^ algorithm works based on degree of collinearity and it is performing better than other meta-heuristic algorithms. However, the disadvantage of this algorithm is that its localization accuracy is in the acceptable range only when the ANs are beyond 20% of the total number of SNs in the network and also the minimum communication distance should be 35 m. DECPSOHDV-Hop algorithm^70– 71^ gives localization accuracy of 84.62% only if the ANs are above 50% of total SNs and the minimum communication range of 50 m. In Fig. 6, localization based on WOA-QT^72^ gives poor localization accuracy than the other algorithms. However, the proposed CERBLA provides localization accuracy of 86.81% when the number of ANs are only four. For the sake of comparison, we have also increased the number of ANs in simulations, and it is observed that the localization accuracy of CERBLA is further increased with increasing number of ANs. The reason to achieve higher localization accuracy with CERBLA is that the selection of more favorable and optimal number of ANs leads to the higher localization accuracy and minimizes energy consumption. Also, the TNs are not dependent on a particular set of ANs instead of the location measurements are performed in a distributed manner and it leads to minimal number of iterations. The localization accuracy using the proposed CERBLA algorithm is enhanced by 25.32%, 25.19%, and 21.47% compared to DCK-GWO, WOA-QT, DECPSODV-Hop algorithms respectively as shown in Table 5.

**Table 5:** Mean localization accuracy (in %) with increasing number of ANs.

Number of ANs																
Protocol used	4	5	6	7	8	9	10	15	20	25	30	35	40	45	50	Average
DCK-GWO	59.14	61.28	64.72	68.18	70.92	73.56	76.54	83.28	88.12	90.04	92.17	92.05	92.85	93.12	93.48	68.12
WOA-QT	53.64	54.52	60.19	62.85	64.88	65.06	65.86	68.78	72.54	73.09	74.47	76.06	76.71	77.24	77.86	68.25
DECPSODV-Hop	63.02	63.24	64.01	64.28	66.71	68.21	71.08	71.18	71.64	72.18	74.04	77.98	83.01	83.98	85.02	72.01
CERBLA (Proposed)	86.81	86.89	87.49	87.57	89.94	90.10	93.61	94.13	95.49	96.81	97.39	98.21	98.94	99.12	99.24	93.44

**Fig. 7:**
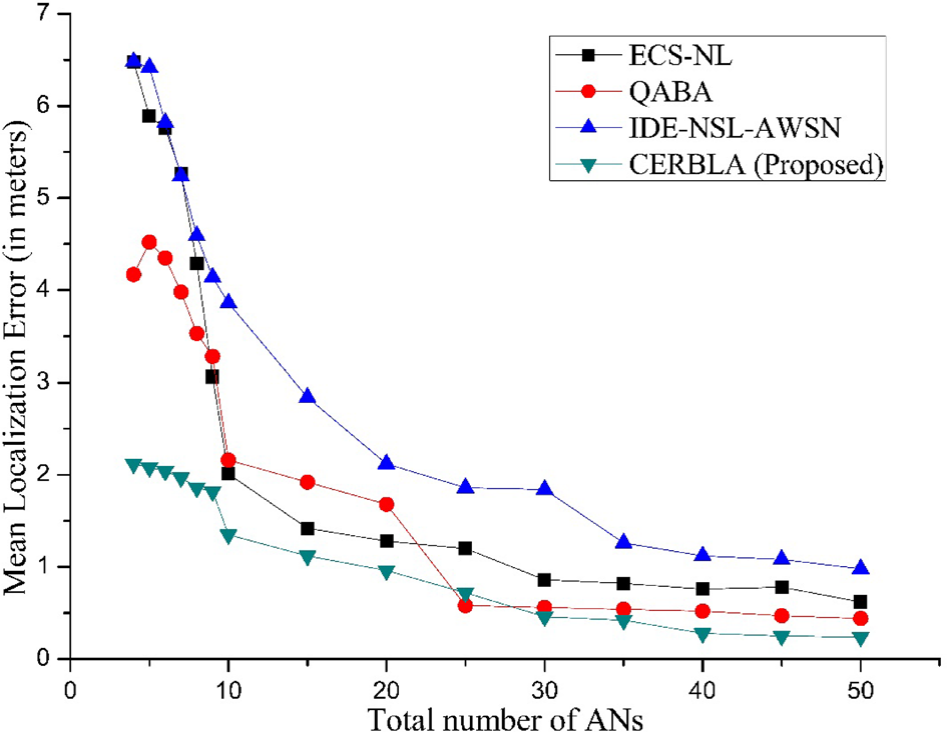
Mean localization error (in meters) vs. ANs in the network.

In Fig. 7, the localization error is measured in meters with increasing number of ANs for different localization algorithms like ECS-NL^73^, QABA^74^, IDE-NSL-AWSN^75^, and compared with CERBLA. When the number of ANs are four, the localization error is 2.12 m using CERBLA and it is higher for other algorithms. In other algorithms, to achieve mean localization error in the range of 2 m, minimum ten ANs are essential which increases the network cost as ANs are associated with GPS modules. Also, the average localization error of CERBLA is limited to 1.18 m when the ANs are varied from 4 to 50. At the same time, the average localization errors of ECE-NL, QABA, and IDE-NSL-AWSN are 2.706 m, 2.18 m, and 3.34 m respectively. The localization error in IDE-NSL-AWSN is more and it expects the minimum communication range of 75 m and 15 m in large-scale and small-scale environments respectively. QABA gives higher localization accuracy when the minimum communication range is 30 m and the average error is limited to 2.18 m. From Table 6, the localization error using the existing localization algorithms is mitigated to minimum levels only after the number of ANs are increased to 10 whereas the need of ANs is limited to four using the proposed algorithm to reach the same error values.

**Table 6:** Mean localization error (in meter) vs. number of ANs.

Number of ANs																
Protocol	4	5	6	7	8	9	10	15	20	25	30	35	40	45	50	Average
ECS-NL	6.48	5.89	5.76	5.26	4.29	3.12	2.14	1.38	1.32	1.18	0.86	0.82	0.76	0.71	0.62	2.706
QABA	4.17	4.52	4.35	3.98	3.53	3.28	2.16	1.92	1.68	0.58	0.56	0.54	0.52	0.47	0.44	2.18
IDE-NSL-AWSN	6.68	6.42	5.82	5.24	4.61	4.24	3.92	2.78	2.44	1.92	1.63	1.26	1.12	1.08	0.98	3.34
CERBLA (Proposed)	2.12	2.08	2.04	1.97	1.86	1.82	1.35	1.12	0.96	0.72	0.46	0.42	0.28	0.25	0.24	1.18

## Conclusions

The aim of this proposed method is to decrease the number of ANs required in the localization process and minimize the cost of the WSN. We have used only four ANs which need GPS modules to know their locations and the rest of the SNs use the signals received from these ANs to locate themselves through CERBLA algorithm. Results of simulations show that the CERBLA algorithm limits the location measurement error to 1.18 m and improves the average localization accuracy up to 99.24%. CERBLA selects more favorable and optimal number of ANs to achieve higher localization accuracy and minimize energy consumption. Also, TNs are independent of ANs set instead the location measurements are performed in a distributed manner and it leads to minimal number of iterations. The localization accuracy using the proposed CERBLA algorithm is enhanced by 25.32%, 25.19%, 21.47%, 128.21%, 84.48%, and 181.35% compared to DCK-GWO, WOA-QT, DECPSODV-Hop, ECS-NL, QABA, and IDE-NSL-AWSN algorithms respectively. Overall, the localization error is minimum using the proposed CERBLA algorithm compared to other algorithms especially when the number of ANs is less than 10. This feature is attractive to build cost-effective localization algorithms for IIoT and other indoor applications of WSNs.

## Supplementary Information

Below is the link to the electronic supplementary material.

Supplementary Material 1

## Data Availability

Data is shared by the corresponding author upon reasonable request.

## Code availability

The custom code is available as supplementary document of the manuscript.

## Glossary

3D: Three dimensional
ABCOA: Artificial bees colony optimization algorithm
ACO: Ant colony optimization
AN: Anchor node
AOA: Angle of arrival
BDS: Beidou navigation satellite system
BOA: Butterfly optimization algorithm
CERBLA: Cost-effective range-based localization algorithm
CRLB: Cramèr–rao lower bounds
CSO: Cuckoo search optimization
DCK: Degree of K-value collinearity
DECPSOHDV: Differential evolution chaotic PSO Hybrid DV
DEEC: Distributed energy efficient clustering
DV: Distance vector
ECS-NL: Enhanced CSA for node localization
FOA: Firefly optimization algorithm
FPO: Fower pollination optimization
FSO: Fish swarm optimization
GA: Genetic algorithm
GPS: Global positioning system
GWO: Grey wolf optimization
IDE-NSL-AWSN: Improving distance estimation based on node selection in obstacle-aware WSNs
IIoT: Industrial internet of things
LOS: Line of sight
LRAPJA: Localization using reliable anchor pair selection-based Jaya algorithm
MCB: Monte carlo localization boxed
ML: Maximum-likelihood
MSE: Mean square error
NLOS: Non-line-of-sight
PSO: Particle swarm optimization
QABA: Quantum annealing bat algorithm
QSSA: Quantum-behaved salp swarm algorithm
QT: Quasiaffine transformation
RAPS: Reliable anchor pair selection
RF: Radio frequency
RSSI: Received signal strength indicator
SN: Sensor node
SNR: Signal-to-noise ratio
TDOA: Time difference of arrival
TLBO: Teaching learning-based optimization
TN: Target node
TOA: Time of arrival
WOA: Whale optimization algorithm
WSN: Wireless sensor network
