# Correction: Optimization of range based self-localization problem in wireless sensor networks using improved cuckoo search algorithm

**DOI:** 10.1038/s41598-026-36522-1

**Published:** 2026-01-21

**Authors:** Srilakshmi Aouthu, Narra Dhanalakshmi, T. Seshagiri, Ravilla Dilli

**Affiliations:** 1 Electronics and Communication Engineering Department, Vasavi College of Engineering, Hyderabad, Telangana 500031 India; 2 Electronics and Communication Engineering, VNR Vignana Jyothi Institute of Engineering & Technology, Hyderabad, Telangana 500090 India; 3 Electronics and Communication Engineering, Sri Venkateswara College of Engineering & Technology, Chittoor, Andhra Pradesh, 517127 India; 4 Electronics and Communication Engineering, Manipal Institute of Technology, Manipal Academy of Higher Education, Manipal, Udupi, Karnataka 576104 India

Correction to: *Scientific Reports* 10.1038/s41598-025-23405-0, published online 13 November 2025

The original version of this Article contained errors.

The name of author Narra Dhanalakshmi was incorrectly given as N. Dhanalakshmi.

In addition, Affiliation 2 was incorrectly given as ‘Electronics and Communication Engineering, VNR Vignan Jyothi Institute of Engineering & Technology, Hyderabad, 500090, Telangana, India’. The correct affiliation is listed below:

Electronics and Communication Engineering, VNR Vignana Jyothi Institute of Engineering & Technology, Hyderabad, 500090, Telangana, India.

The original Article has been corrected.

