# Supplementary material for: Optimization of range based self-localization problem in wireless sensor networks using improved cuckoo search algorithm
Source: Sci Rep. 2025 Nov 13;15:39803. doi: 10.1038/s41598-025-23405-0 (PMC12615830; doi:10.1038/s41598-025-23405-0)
Supplement: Supplementary file 1 — Supplementary Material 1 [file 41598_2025_23405_MOESM1_ESM.pdf]

```
N = 4;
M = 100;
distMeasurementErrRatio = 0.01;
networkSize = 100;
anchorLoc   = [25   25;  75   25;  25   75;  75   75];
mobileLoc  = networkSize*rand(M,2);
distance = zeros(N,M);
 for m = 1 : M
    for n = 1 : N
           distance(n,m) = sqrt( (anchorLoc(n,1)-mobileLoc(m,1)).^2 + ...
                                   (anchorLoc(n,2)-mobileLoc(m,2)).^2  );
    end
 end
 f1 = figure(1);
 clf
 plot(anchorLoc(:,1),anchorLoc(:,2),'go','MarkerSize',12,'lineWidth',2,'MarkerFaceColor','r');
 grid on
 hold on
 plot(mobileLoc(:,1),mobileLoc(:,2),'b+','MarkerSize',8,'lineWidth',2);
 distanceNoisy = distance + distance.*distMeasurementErrRatio.*(rand(N,M)-1/2);
  numOfIteration = 5;
  mobileLocEst = networkSize*rand(M,2);
  for m = 1 : M
    for i = 1 : numOfIteration
```

```
        distanceEst   = sqrt(sum( (anchorLoc - repmat(mobileLocEst(m,:),N,1)).^2 , 2));

        distanceDrv   = [(mobileLocEst(m,1)-anchorLoc(:,1))./distanceEst ...

                    (mobileLocEst(m,2)-anchorLoc(:,2))./distanceEst];

         delta = - (distanceDrv.'*distanceDrv)^-1*distanceDrv.' * (distanceEst -
distanceNoisy(:,m));

        mobileLocEst(m,:) = mobileLocEst(m,:) + delta.';

      end

   end

   plot(mobileLocEst(:,1),mobileLocEst(:,2),'go','MarkerSize',8,'lineWidth',2);

   legend('AN location','TN Actual location','TN Estimated location',...

        'Location','Best')

   Err = mean(sqrt(sum((mobileLocEst-mobileLoc).^2)));

   title(['Mean Estimation error is ',num2str(Err),'meter'])

   axis([-0.1 1.1 -0.1 1.1]*networkSize)
```
